# Prevalence of *KRAS*, *BRAF*, and *PIK3CA* somatic mutations in patients with colorectal carcinoma may vary in the same population: clues from Sardinia

**DOI:** 10.1186/1479-5876-10-178

**Published:** 2012-08-29

**Authors:** Grazia Palomba, Maria Colombino, Antonio Contu, Bruno Massidda, Giovanni Baldino, Antonio Pazzola, MariaTeresa Ionta, Francesca Capelli, Vittorio Trova, Tito Sedda, Giovanni Sanna, Francesco Tanda, Mario Budroni, Giuseppe Palmieri, Antonio Cossu

**Affiliations:** 1 Istituto di Chimica Biomolecolare, CNR, Sassari, Italy; 2 Servizio Oncologia, ASL1, Sassari, Italy; 3 Oncologia Medica, Università di Cagliari, Cagliari, Italy; 4 Oncologia, ASL, Nuoro, Italy; 5 Oncologia, ASL, Alghero, Italy; 6 Oncologia, ASL, Oristano, Italy; 7 Oncologia Medica, AOU, Sassari, Italy; 8 Anatomia Patologica, Università di Sassari, Sassari, Italy; 9 Servizio Epidemiologia, ASL1, Sassari, Italy; 10 Anatomia Patologica, AOU, Sassari, Italy; 11 Unit of Cancer Genetics, Institute Biomolecular Chemistry (ICB), National Research Council (CNR), Traversa La Crucca 3, Loc. Baldinca Li Punti, Sassari 07100, Italy

**Keywords:** Colorectal carcinoma, *KRAS* gene, *BRAF* gene, *PIK3CA* gene, Mutation analysis, Cancer genetic heterogeneity

## Abstract

**Background:**

Role of *KRAS*, *BRAF* and *PIK3CA* mutations in pathogenesis of colorectal cancer (CRC) has been recently investigated worldwide. In this population-based study, we evaluated the incidence rates and distribution of such somatic mutations in genetically isolated population from Sardinia.

**Methods:**

From April 2009 to July 2011, formalin-fixed paraffin-embedded tissues (N = 478) were prospectively collected from Sardinian CRC patients at clinics across the entire island. Genomic DNA was isolated from tissue sections and screened for mutations in *KRAS*, *BRAF*, and *PIK3CA* genes by automated DNA sequencing.

**Results:**

Overall, *KRAS* tumour mutation rate was 30% (145/478 positive cases). Distribution of mutation carriers was surprisingly different within the island: 87/204 (43%) in North Sardinia *vs.* 58/274 (21%) in Middle-South Sardinia (p<0.001). Among 384 CRC cases whose DNA was available, only one (0.3%) patient carried a mutation in *BRAF* gene; *PIK3CA* was found mutated in 67 (17%) patients. A significant inverse distribution of *PIK3CA* mutation rates was observed within Sardinian population: 19/183 (10%) cases from northern *vs.* 48/201 (24%) cases from central-southern island (p<0.001). This heterogeneity in frequencies of *KRAS*/*PIK3CA* somatic mutations is consistent with already-reported discrepancies in distribution of germline mutations for other malignancies within Sardinian population. Preliminary clinical evaluation of 118 *KRAS* wild-type patients undergoing anti-EGFR-based treatment indicated lack of role for *PIK3CA* in predicting response to therapy.

**Conclusions:**

Our findings support the hypothesis that differences in patients’ origins and related genetic backgrounds may contribute to even determine the incidence rate of somatic mutations in candidate cancer genes.

## Introduction

Colorectal carcinoma (CRC) develops through different pathways, all involving changes at the chromosomal or gene levels. It is now widely accepted that sporadic colorectal cancers frequently arise from preneoplastic lesions through the activation of oncogenes (*KRAS* and *BRAF*) as well as the inactivation of tumour suppressor genes (*APC, p16, p53*, and *DCC*) and mismatch repair genes, such as *MLH1* and *MSH2* and, to a lower extent, *PMS2* and *hMSH6*[1]. In addition, activating mutations in *BRAF*, a member of the *RAF* gene family, which encode kinases that are regulated by members of the RAS protein family (HRAS, KRAS, and NRAS) and mediate cellular responses to growth signals, were found to be associated with microsatellite instability (MSI) cancers [2,3]. As stated above, *KRAS* is the member of the *RAS* gene family mostly mutated in CRC; unlike the *BRAF* mutations, the *KRAS* mutations have been found to be equally distributed in all tumours, regardless of their MSI status [4]. These findings acquire an important role from the pathogenetic point of view since the mutations that hit these two genes are reciprocally exclusive. On such a basis, the occurrence of an activating mutation at either one of the two genes may be linked to different molecular processes and, therefore, may generate at least three distinct tumour subtype: *BRAF*^mut^/MSI+, *KRAS*^mut^/MSI+, and *KRAS*^mut^/MSI- [2-4].

Mutation of *KRAS* is an established predictor of absence of response to epidermal growth factor receptor (EGFR)-targeted agents [5]. The utility of *KRAS* as a prognostic marker remains uncertain. A recent meta-analysis reported that *KRAS* mutation may act as a negative prognostic indicator in both a trans-stage and stage-specific setting [6], whereas other studies, such as the large PETACC-3 translational trial [7], reported the absence of any prognostic value. On this regard, survival from recurrence was markedly worse in *BRAF*-mutant tumours into the PETACC-3 trial and this observation was consistent with the previously reported poor prognosis of *BRAF* mutations in advanced (stage IV) CRCs [8]. Unlike *KRAS* mutations, *BRAF* mutations might not be predictive of lack of anti-EGFR therapy benefit [3]. Among these genes, nearly all mutations affect the kinase domains at codons 12 or 13 of *KRAS* and codon 600 of *BRAF*[3,9].

In addition to the RAS/RAF/MEK pathway, the phosphatidylinositol 3-kinase (PI3K)/AKT/mTOR signaling cascade does participate in regulating cell proliferation and survival, apoptosis, and migration [10]. Activation of the PI3K/AKT/mTOR pathway is frequently mediated by mutations in the p110α subunit of *PI3K, PIK3CA*, with most mutations (>80%) occurring either in exon 9, which codes for the helical domain, or exon 20, which codes for the kinase domain [10].

In population-based studies, the prevalence of *KRAS, BRAF*, and *PIK3CA* mutations ranges from 30% to 40% for *KRAS* mutations, from 5% to 15% for *BRAF* mutations, and from 10% to 15% for *PIK3CA* mutations [11]. Somatic mutations of *PIK3CA* may coexist with either *KRAS* or *BRAF* mutations within the same tumor [12], but *KRAS* and *BRAF* mutations appear to be mutually exclusive [13].

In Sardinia, which has experienced little immigration due to its remote location and whose population has inherited many of the same genetic traits, the contribution of somatic mutations in these three genes to the CRC pathogenesis has not been estimated yet. Colorectal cancer represents the second principal death-causing malignancy in Sardinia, with an incidence (standardized rate, 104 per 100.000 inhabitants per year; Sardinian population includes about one million and half inhabitants) quite comparable with that observed in Western countries [14].

As previously demonstrated by our group for other malignancies (mainly, breast cancer and malignant melanoma), the geographical distribution of germline sequence variants across the island seems to be significantly heterogeneous, suggesting that the genetic background may influence the occurrence of cancer gene mutations [15-17]. In this study, we assessed the prevalence and distribution of *KRAS, BRAF*, and *PIK3CA* mutations at somatic level among 478 consecutively-collected CRC patients from Sardinian population.

## Materials and methods

### Samples

Four hundred and seventy-eight patients with histologically-proven diagnosis of colorectal carcinoma (CRC) and regularly participating to the follow-up programs at the Institutions across Sardinia island were included into the study. To avoid any bias, CRC patients were consecutively collected from April 2009 to September 2011; they were included regardless of age at diagnosis and disease characteristics. No CRC case from our series was associated with clinically relevant colorectal polyposis. Sardinian origin was ascertained in all cases through genealogical studies; for all patients, place of birth of their parents and grandparents was assessed in order to assign their geographical origin within the island. Clinical and pathological features for the assessment of the disease stage at diagnosis as well as of the onset age and tumour anatomical location were confirmed by medical records and/or pathology reports. Disease stage classification was assigned according to the American Joint Committee on Cancer guidelines [18].

Formalin-fixed paraffin embedded tissue samples from CRC patients were obtained from the archives of the Institutes and Services of Pathology participating to the study. Tissue samples were estimated to contain at least 70% neoplastic cells by light microscopy.

All patients were informed about the aims of this study and, before the tissue sample was collected, gave a written informed consent. The study was reviewed and approved by the ethical review board of the University of Sassari.

### Mutation analysis

All tumour tissues were collected and processed at the laboratory of the Institute of Biomolecular Chemistry of Sassari; genomic DNA was isolated from tissue sections using a standard protocol and DNA quality assessed for each specimen. In particular, paraffin was removed from formalin-fixed paraffin-embedded (FFPE) samples by treatment with Bio-Clear (Bio-optica, Milan, Italy) and DNA was purified using the QIAamp DNA FFPE Tissue kit (QIAGEN Inc., Valencia, CA, USA).

The coding sequence and splice junctions of exons 2 and 3 in *KRAS* gene (where all pathogenetic mutations occur [9]), exon 15 in *BRAF* gene (nearly all oncogenic mutations have been detected at the kinase domain in exon 15 [19,20]), and exons 9 and 20 in *PIK3CA* gene (they represent the mostly mutated domains of this gene [10,21]) were screened for mutations by direct automated sequencing. Briefly, polymerase chain reaction (PCR) was performed on 25–50 ng of isolated genomic DNA in a 9700 Thermal cycler (Applied Biosystems, Foster City, CA, USA); all PCR-amplified products were directly sequenced using an automated fluorescence-based cycle sequencer (ABIPRISM 3100, Applied Bio-systems, Foster City, CA), as previously described by our group [17]. Primer sequences were as follow: *KRAS* exon 2 forward, TGTGTGACATGTTCTAATATAGTCACAT - exon 2 reverse, GGTCCTGCACCAGTAATATGC - exon 3 forward, GACTGTGTTTCTCCCTTCT - exon 3 reverse, TGGCAAATACACAAAGAAAG; *PIK3CA* exon 9 forward, GGGAAAAA TATGACAAAGAAAGC - exon 9 reverse, CTGAGATCAGCCAAATTCAGTT - exon 20 forward, CTCAATGATGCTTGGCTCTG - exon 20 reverse, TGGAATCCAGAGTGAGC TTTC; *BRAF* exon 15 forward, TCATAATGCTTGCTCTGATAGGA - exon 15 reverse, GGCCAAAAATTTAATCAGTGGA. Protocols for PCR-based assays were designed and optimized in our laboratory; they will be available upon request. Screening for *BRAF* and *PIK3CA* genes was incomplete in a fifth of patients (94/478; 20%) due to the low amount of available tumour tissue samples.

### Statistical analysis

Statistical analysis for the presence of *KRAS, BRAF,* or *PIK3CA* mutations versus different variables (sex, age at diagnosis, anatomical site of primary CRC, disease stage, geographical origin of patients) was performed by Pearson’s Chi-Square test. The odds ratio (OR) and 95% confidence interval (CI) values were calculated by logistic regression analysis. The exact coefficient for sample proportion analysis was performed to determine all significant parameters (below 0.05 level). All analyses were performed using the statistical package SPSS/7.5 per Windows.

## Results

Paraffin-embedded tumour tissues from a total of 478 patients with advanced colorectal carcinoma (CRC) originating from different geographical areas within Sardinia island were screened for mutations in candidate genes. Considering the primary tumour, left colon was the most frequent anatomical location (left colon, 192 [40%]; right-transverse colon, 172 [36%]; rectum, 114 [24%]) (Table 1). The median age was 64 years (range, 31–87 years), with a preponderance of males (291 men; 61%). At the time of diagnosis, minority of patients presented with localized disease (AJCC stage II, 173 [36%] versus AJCC advanced stages III and IV, 160 [34%] and 145 [30%], respectively) (Table 1).

**Table 1:** Distribution of mutations according to the characteristics of CRC patients

***Characteristic***		***KRAS*mut**		***PI3K*mut**
	***No. (N = 478)***		***No. (N = 384)***	
**Sex**				
Male	*293*	84 (29%)	*231*	38* (16%)
Female	*185*	61 (33%)	*153*	29 (19%)
**Tumor site**				
Right-transverse colon	*172*	54 (31%)	*138*	25* (18%)
Left colon	*192*	59 (31%)	*151*	23 (15%)
Rectum	*114*	32 (28%)	*95*	19 (20%)
**Disease stage**				
Stage II	*173*	44 (25%)	*143*	22 (15%)
Stage III	*160*	56 (35%)	*128*	25 (20%)
Stage IV	*145*	45 (31%)	*113*	20* (18%)
**Tumor grading**				
Well differentiated	*56*	17 (30%)	*44*	6 (14%)
Moderately differentiated	*383*	118 (31%)	*309*	54 (17%)
Poorly differentiated	*39*	10 (26%)	*31*	7* (23%)
**Age, years**				
< 50	*50*	16 (32%)	*45*	5 (11%)
50-59	*116*	38 (33%)	*102*	14 (14%)
60-69	*178*	56 (31%)	*151*	30* (20%)
70+	*134*	35 (26%)	*86*	18 (21%)

* 1 patient also mutated in BRAF.

**Figure 1:**
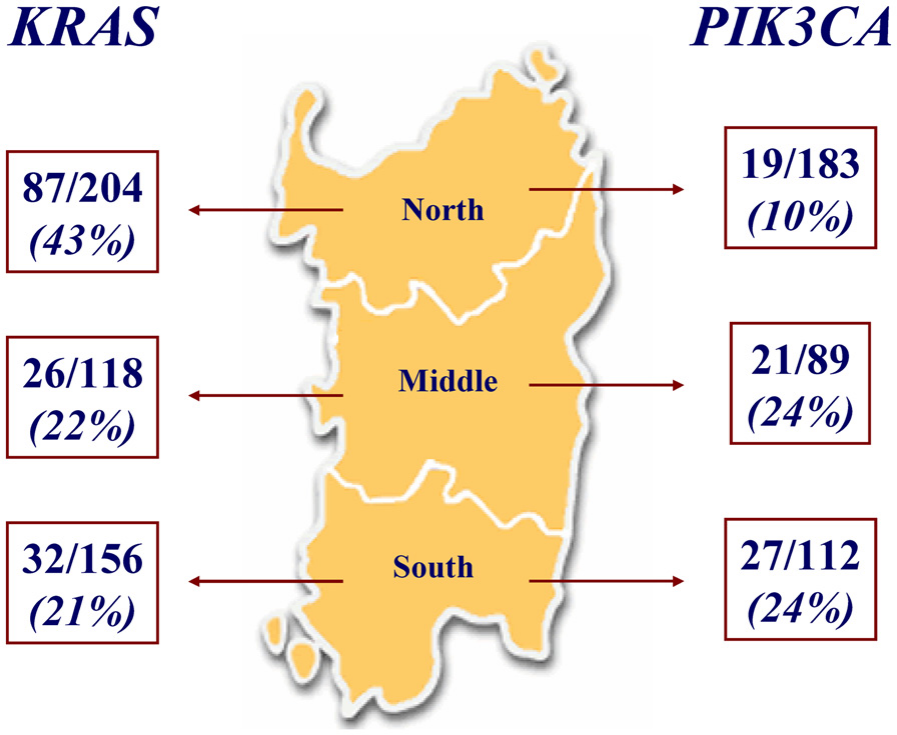
**Frequencies of*KRAS*and*PIK3CA*mutations across Sardinia.** The three geographical regions within the island are indicated.

The full coding sequences and intron-exon junctions of the *KRAS* gene were sequenced in the entire series of 478 CRC patients; *KRAS* mutations were detected in 145 (30%) primary tumours (one patient had two mutations, G12D and Q61L). In terms of the gene positions of the identified *KRAS* mutations, 73% (N = 106) of them affected codon 12 and 20% (N = 29) affected codon 13, whereas the remaining 7% (N = 11) affected other codons (mainly, codon 61) (Table 2). All *KRAS* mutations detected in the present study have been previously reported in the Human Gene Mutation Database (HGMD) [22] and in the Catalogue Of Somatic Mutations In Cancer (COSMIC) [23]. Considering the patients’ origin within the Sardinia island, distribution of mutations was significantly heterogeneous: 87/204 (43%) mutated cases in North Sardinia versus 58/274 (21%) in Middle-South Sardinia [p<0.001 (OR, 2.82; 95% CI, 2.75-2.89); p normalized scientific notation = 6.8 x 10^-4^ (Figure 1). Such discrepancies did not result from incorrect standard sequencing as confirmed by an independent duplicate analysis. 

**Table 2:** **Somatic mutations in*KRAS*gene**

**Mutation**	**No.**	***%***
***Codon 12***		
**G12A**	13	*9*
**G12C**	11	*8*
G12D*	41	*28*
**G12R**	4	*3*
**G12S**	5	*3*
**G12V**	32	*22*
**Total**	106	*73*
***Codon 13***		
**E49K**	1	*0.5*
***Codon 49***		
**G13C**	4	*3*
**G13D**	23	*16*
**G13S/V**	2	*1*
**Total**	29	*20*
***Codon 59***		
**Q61R**	2	*1*
Q61L*	7	*5*
**Total**	9	*6*
***Codon 61***		
**A59E**	1	*0.5*

Percentages are referred to mutation frequencies among the 145 positive cases.

* 1 patient with two mutations.

Among available DNA samples, 384 primary tumours were also evaluated for occurrence of *BRAF* (in exon 15) and *PIK3CA* (in exons 9 and 20) mutations. Overall, mutations were detected in 1 (0.3%) patient for *BRAF* gene and 67 (17.4%) cases for *PIK3CA* gene.

The single BRAF-mutated patient presented: *a*) the substitution of valine by a glutamic acid at position 600 (V600E), which has been demonstrated to account for majority (about 90%) of the *BRAF* mutations identified [19,20,24]; and *b*) a concomitant *PIK3CA* mutation, whereas no *KRAS* mutation coexisted. For *PIK3CA* mutations, screening revealed the occurrence of six mutations (p.E542K, p.E545A, p.E545G, and p.E545K in exon 9; p.M1043I and p.H1047R in exon 20), which have been widely reported in mutation databases (HGMD and COSMIC; see above) as commonly associated with CRC, with a recognized functional role of the corresponding mutated proteins. The variant p.E545A was the mutation with the highest frequency in our series (detected in 54/384 [14%] cases) (Table 3). Mutations of *PIK3CA* and *KRAS* were found to coexist in 15/384 (3.9%) of cases. 

**Table 3:** **Prevalence of somatic mutations in*PIK3CA*gene**

**Exon**	**Mutated cases**(%)*	**Protein**	**DNA**
9	1 *(1.5%)*	E542K	c.1624 G > A
	54 *(80.6%)*	E545A	c.1634A > C
	4 *(6.0%)*	E545G	c.1634A > G
	1 *(1.5%)*	E545K	c.1633 G > A
20	1 *(1.5%)*	M1043I	c.3129 G > T
	6 *(8.9%)*	H1047R	c.3140A > G

*percentages are referred to the series of 67 positive cases.

Table 4 summarizes the distribution and relationship of the somatic mutations identified in the series of 384 CRC tumours for all three candidate genes. Altogether, a mutation of at least one gene was discovered in about half (174/384; 45.3%) of CRC cases; in other words, 54.7% (N = 210) primary tumours displayed a wild-type genetic status in these three genes (Table 4).

**Table 4:** Frequencies of somatic mutations in the series of 384 patients screened for all three genes, according to the geographical origin

***Patients’ origin***	***KRAS***			***PIK3CA***	***wild-type***
		***KRAS + PIK3CA***	***BRAF + PIK3CA***		
	***%***	***%***	***%***	***%***	***%***
**North Sardinia** (N = 183)	**66**	**8**	**0**	**11**	**98**
	*36.1*	*4.4*	*0*	*6.0*	*53.5*
**Middle-South Sardinia** (N = 201)	**41**	**7**	**1**	**40**	**112**
	*20.4*	*3.5*	*0.5*	*19.9*	*55.7*
**Total** (N = 384)	**107**	**15**	**1**	**51**	**210**
	*27.8*	*3.9*	*0.3*	*13.3*	*54.7*

**Figure 2:**
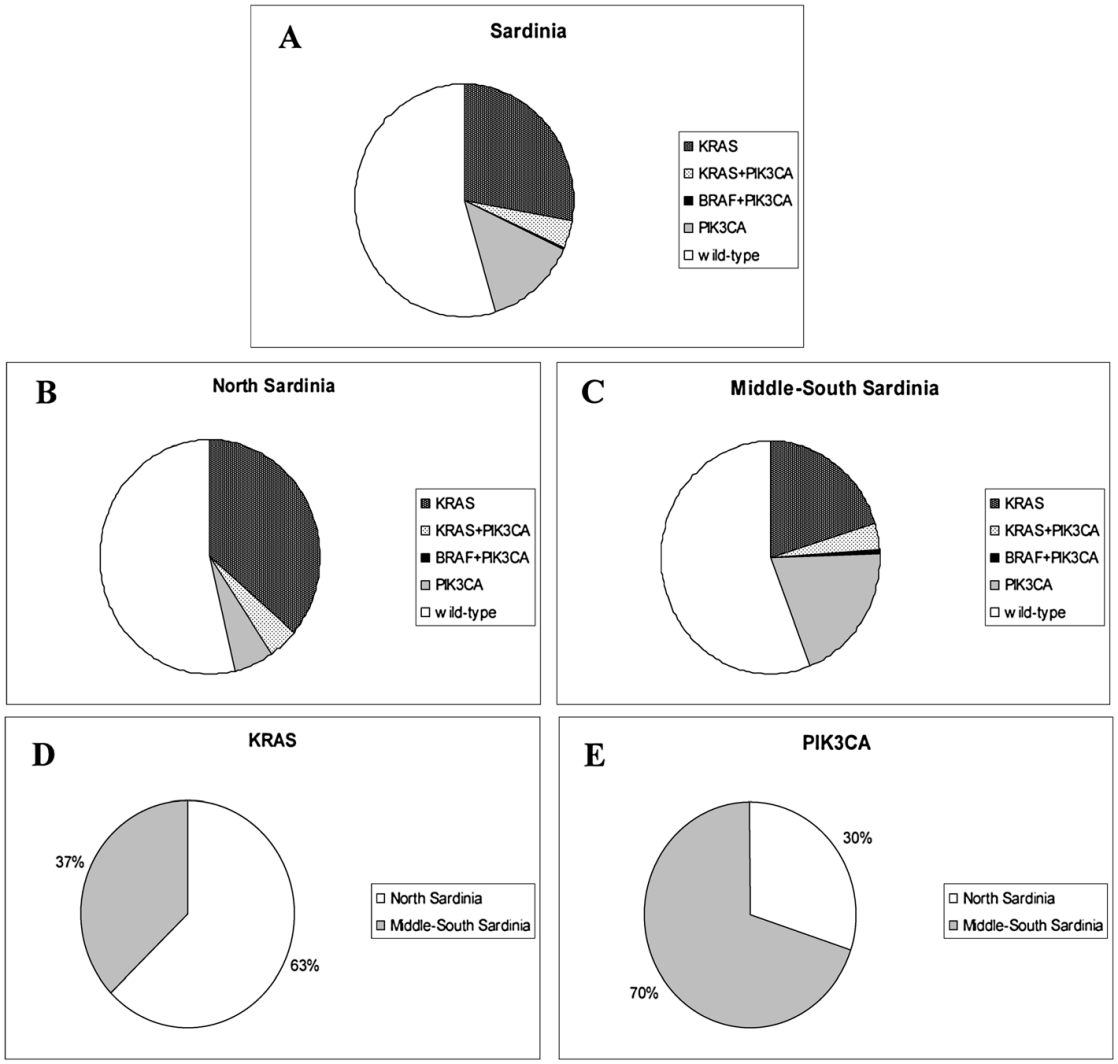
**Geographical distribution of mutation carriers in the series of 384 patients screened for all three genes.** (**A**) Entire island. (**B**) Northern and (**C**) Central-Southern regions. Prevalence of (**D**) *KRAS*- and (**E**) *PIK3CA*-mutated patients according to the geographical origin.

Considering the patients’ origin, *PIK3CA* mutations were found to be inversely distributed as compared to the *KRAS* mutations: 19 (10%) out of 183 patients from North Sardinia versus 48 (24%) out of 201 patients from Middle-South Sardinia were found to carry mutations in exons 9 and 20 of the *PIK3CA* gene (Table 4; Figure 1). As for *KRAS*, such a heterogeneous distribution of *PIK3CA* mutations was found highly significant [p<0.001 (OR, 2.45; 95% CI, 2.36-2.55); p normalized scientific notation = 5.9 x 10^-4^]. To avoid any putative artefact, such discrepancies were again confirmed in independent duplicate sequencing experiments. As shown in Figure 2, majority (63%) of *KRAS* mutations were found in patients from North Sardinia whereas more than two thirds (70%) of *PIK3CA* mutations were detected in patients from Middle-South Sardinia. No difference in distribution of *KRAS* and *PIK3CA* mutations between rural and urban areas, both globally and within the two (North *vs.* Middle-South) geographical regions was observed.

Both *KRAS* and *PIK3CA* mutations were evaluated for association with several pathological parameters: sex, age at diagnosis, anatomical location of primary CRC, tumour grading, AJCC stage of the disease. In our series, no significant correlation was found between the occurrence of *KRAS* or *PIK3CA* mutations and all analyzed parameters. However, a non-significant trend for *PIK3CA* mutations to be associated with a older age of onset and a higher tumour grade was observed (see Table 1). Absence of any association between the different variables was also confirmed by a multivariate analysis.

Finally, we preliminarily evaluated the association between *PIK3CA* mutations and response to the treatment with anti-EGFR monoclonal antibodies in patients with wild-type *KRAS*. Among the 118 *KRAS* mutation negative patients for whom results were available (105 [89%] treated with cetuximab in association with systemic chemotherapy and 13 [11%] with panitumumab alone), the objective response rate at first evaluation was 30% (N = 35; no complete clinical response was registered) (Table 5). In this subset of patients, a slight but not significant difference in rates of partial responses was observed between *PIK3CA*-mutated (7/29; 24%) and *PIK3CA*-wild-type (28/89; 31%) cases (Table 5).

**Table 5:** **Clinical response to anti-EGFR therapy in*KRAS*wild-type patients**

		***KRAS*wt**	
	***KRAS*wt (N = 118)**	***PI3K*wt (N = 89)**	***PI3K*mut (N = 29)**
**PR**	35 (30%)	28 (31%)	7 (24%)
**SD**	58 (49%)	45 (51%)	13 (45%)
**PD**	25 (21%)	16 (18%)	9 (31%)

*PR*, partial response; *SD*, stable disease; *PD*, progression disease. *wt*, wild-type.

## Discussion

In this study, we have reported the prevalence of somatic mutations in *KRASPIK3CA*, and *BRAF* genes among patients with advanced colorectal carcinoma from Sardinia, whose population shows genetic peculiarity due to geographical isolation and strong genetic drift [25]. Prevalence of candidate gene mutations may vary among distinct populations due to concurrence of different environmental factors and genetic backgrounds. Furthermore, patients’ geographical origin within the same population may also account for different mutation rates in pathogenetic cancer genes, as already demonstrated for different types of cancer in Sardinian population by our group [15-17]. In summary, we observed a moderate rate of *KRAS* mutations (145/478; 30.3%) or *PIK3CA* mutations (67/384; 17.4%) and a very low rate of *BRAF* mutations (1/384; 0.3%) in a cohort of primary colorectal carcinomas.

Prevalence of *KRAS* mutations in our series is consistent with data from literature, indicating that such an alteration can be found in 30-40% of colorectal carcinomas [6]. Controversial data have been instead published about the *PIK3CA* mutation rates, ranging from 7% [26] to 30% [27] of CRC cases presenting a mutated *PIK3CA*. In the present study, all detected *PIK3CA* variants have been previously demonstrated to be oncogenic in CRC cellular models [28] and commonly associated with colorectal carcinoma (17%; see Table 3); the mutation rate was therefore comparable with that described in majority of previous reports.

The most surprising finding was the nearly lack of *BRAF* mutations in our series. In recent meta-analyses, the BRAF V600E mutation - which represents the most common mutation in *BRAF* gene (more than 90% of cases) - was detected in about 9% of primary colorectal carcinomas [29,30]. One could speculate that the very low frequency of *BRAF* mutation detected in our series may be somehow due to patients’ origin or, in other words, to the peculiarity of the genetically-isolated Sardinian population. On this regard, it cannot be excluded that different pathogenetic mechanisms of transformation could occur in different populations. Microsatellite instability (MSI), a recognized marker of a tendency for replication errors in human cancers, has been widely indicated as a factor associated with higher frequency of mutations in *BRAF* gene among colorectal carcinomas [29,31,32]. Although such an analysis was not conducted in the present study, Sardinian CRC population has been previously demonstrated to present an incidence of MSI similar to that observed in other CRC populations from Western countries [33,34]. Therefore, this factor could not explain the striking discrepancy on the *BRAF* mutation prevalence in our series.

Considering the two prevalent alterations, *KRAS* and *PIK3CA* mutations were more or less equally distributed among the different patients’ subsets, and no statistically significant correlation with sex, onset age (though patients with older age at diagnosis and a higher tumour grade are more likely to present with a *PIK3CA* mutation), disease stage, primary CRC location, or tumour grading was observed (see Table 1).

As schematically represented in Figure 2, we made comparisons between prevalence of *KRAS* and *PIK3CA* mutations within different geographical areas of the island. In a population sharing a quite similar lifestyle and diet habit across the island (moreover, smokers were homogeneously distributed among patients of different origin - though such an information from medical records was available in only about 70% of cases from our series), the observation that a higher frequency of *KRAS* mutations was found in CRC cases from North Sardinia (43% *vs.* 21%) whereas a higher prevalence of *PIK3CA* mutations was found in patients from South Sardinia (24% *vs.* 10%) strongly suggest that different “genetic background” may also induce discrepant penetrance and distribution of somatic mutations in candidate cancer genes. As for similar data reported by our group in breast cancer and melanoma, the geographical distribution of the genetic variants in the island seems to be related to the specific large areas of Sardinia, which reflect its ancient history: the North area, delimited by the mountain chain crossing Sardinia and linguistically different from the rest of the island; and the Middle-South area, land of the ancient Sardinian population and domain of pastoral culture. Nevertheless, our findings clearly indicate that mutation frequency for any candidate cancer gene needs to be accurately evaluated in each geographical area within every single population. Due to this unexpected heterogeneity in distribution of somatic mutations in such few main genes, we already started the collection of tumour DNA samples from Sardinian colorectal cancer patients in order to perform a whole-exome sequencing and define a more comprehensive pattern of mutations in this population.

Finally, although performed on a limited number of CRC cases, our preliminary data seemed to indicate no correlation between *PIK3CA* mutations and response to the anti-EGFR treatment in patients with wild-type *KRAS* (objective responses were considered at first evaluation only) (see Table 5). A second study focused on evaluating such clinical aspects in a larger subset of patients is ongoing.

*KRAS* mutations are considered as an early event in the sequential accumulation of molecular alterations underlying the progression from colorectal adenoma to malignant carcinoma, resulting in an important tumour growth advantage. During the recent past years, a targeted therapy with monoclonal antibodies (cetuximab and panitumumab), blocking the EGFR-driven cell proliferation signals, has been introduced into the therapy of metastatic colorectal cancer [9,35]. No significant response to therapy with anti-EGFR antibodies have been observed in colorectal cancer patients exhibiting *KRAS* mutations [36]. Moreover, majority of colorectal carcinomas exhibiting wild-type *KRAS* do not respond to such therapies either [9]. These phenomena are expected from the molecular point of view, since EGFR tyrosine kinase transmits proliferation signals via RAS-GTPase on the cell membrane inner surface, which in turn can bind effector proteins such as RAF or PIK3CA kinases [37]. Therefore, the occurrence of functional integrity of the RAS-driven pathways - BRAF-MEK-ERK and PIK3CA-AKT - is necessary in order to really interfere with tumour cell growth through inhibition of EGFR target. In other words, the assessment of mutational status of *BRAF* and *PIK3CA* genes into the *KRAS* wild-type population may indeed improve the selection of patients presenting such a functional integrity of the RAS-driven pathways (though we are aware that additional alterations in downstream effectors may intervene). In contrast to *KRAS*, the heterogeneity of *BRAF* and *PIK3CA* mutations has not been adequately investigated in colorectal cancer thus far.

In the present study, because some somatic mutations (in *BRAF* and *PIK3CA* genes or in *KRAS* and *PIK3CA* genes) occurred concomitantly in a given patient, which is in line with literature, a total of about 45% of all patients showed at least one mutation in any of these three genes (see Table 4). Therefore, our data suggest that including mutation analyses for *BRAF* and *PIK3CA* in addition to *KRAS* into a standard diagnostic setting of colorectal cancer would allow the identification of an additional fraction (in our case, about 15%) of patients who cannot be considered as “true wild-type” for such main proliferation-controlling genes. However, whether or not these additional patients might benefit from EGFR-specific antibody therapy has to be verified in prospective clinical studies.

## Conclusions

Although Sardinian population is considered genetically homogeneous, the results obtained in the present study may represent a clear indication that: *a*) differences into the genetic background - related to distinct patients’ origin within the island - may account for different mutation rates in candidate cancer genes (in our series, *KRAS* and *PIK3CA*), even at somatic level; and *b*) mutation frequency for any candidate cancer gene needs to be accurately evaluated in each geographical area.

## Abbreviations

COSMIC: Catalogue of somatic mutations in cancer; CRC: Colorectal carcinoma; EGFR: Epidermal growth factor receptor; FFPE: Formalin-fixed paraffin-embedded; HGMD: Human gene mutation database; MSI: Microsatellite instability; PCR: Polymerase chain reaction.

## Competing interests

The authors declare that they have no competing interests.

## Authors’ contributions

GPa, performed mutation analysis, data analysis and interpretation, helped to draft the manuscript; MC, carried out mutation analysis; ACon, BM, GB, AP, MI, FC, VT, TS, and GS participated in patients' collection and data acquisition; FT, performed quality control of pathological data; MB, performed statistical analysis; GPi, performed data analysis and interpretation, participated in the design of the study, drafted the manuscript; Acos, performed pathological review and data interpretation, conceived of the study. All authors read and approved the final manuscript.
